# Empiric antibiotic, mechanical ventilation, and central venous catheter duration as potential factors mediating the effect of a checklist prompting intervention on mortality: an exploratory analysis

**DOI:** 10.1186/1472-6963-12-198

**Published:** 2012-07-13

**Authors:** Curtis H Weiss, Stephen D Persell, Richard G Wunderink, David W Baker

**Affiliations:** 1 Division of Pulmonary and Critical Care Medicine, Northwestern University Feinberg School of Medicine, Suite 1400 676 N. St. Clair, Chicago, 60611, IL, USA; 2 Division of General Internal Medicine, Northwestern University Feinberg School of Medicine, 10th floor 375 East Chicago Ave, Chicago, IL, 60611, USA

**Keywords:** Empiric antimicrobial agents, Quality improvement, Critical care outcomes, Checklist

## Abstract

**Background:**

Checklists are clinical decision support tools that improve process of care and patient outcomes. We previously demonstrated that prompting critical care physicians to address issues on a daily rounding checklist that were being overlooked reduced utilization of empiric antibiotics and mechanical ventilation, and reduced risk-adjusted mortality and length of stay. We sought to examine the degree to which these process of care improvements explained the observed difference in hospital mortality between the group that received prompting and an unprompted control group.

**Methods:**

In the medical intensive care unit (MICU) of a tertiary care hospital, we conducted face-to-face prompting of critical care physicians if processes of care on a checklist were being overlooked. A control MICU team used the checklist without prompting. We performed exploratory analyses of the mediating effect of empiric antibiotic, mechanical ventilation, and central venous catheter (CVC)duration on risk-adjusted mortality.

**Results:**

One hundred forty prompted group and 125 control group patients were included. One hundred eighty-three patients were exposed to at least one day of empiric antibiotics during MICU admission. Hospital mortality increased as empiric antibiotic duration increased (*P<0*.001). Prompting was associated with shorter empiric antibiotic duration and lower risk-adjusted mortality in patients receiving empiric antibiotics (OR 0.41, 95% CI 0.18-0.92, *P=*0.032). When empiric antibiotic duration was added to mortality models, the adjusted OR for the intervention was attenuated from 0.41 to 0.50, suggesting that shorter duration of empiric antibiotics explained 15.2% of the overall benefit of prompting. Evaluation of mechanical ventilation was limited by study size. Accounting for CVC duration changed the intervention effect slightly.

**Conclusions:**

In this analysis, some improvement in mortality associated with prompting was explained by shorter empiric antibiotic duration. However, most of the mortality benefit of prompting was unexplained.

## Background

Checklists are clinical decision support tools that improve patient care [1-5]. We have previously shown that prompting physicians to use a daily rounding checklist was associated with a reduced risk-adjusted mortality odds ratio of 0.34 and reduced risk-adjusted ICU length of stay [6]. The intervention was associated with several process of care improvements, including reduced empiric antibiotic utilization, that may have contributed to these benefits [6]. Empiric antibiotic therapy—defined as antibiotics administered without culture-documented infection—is frequently administered even when the likelihood of infection is low [6,7]. Overuse of empiric antibiotics can lead to super infection or antibiotic resistance, and may increase mortality [8,9]. Even in study populations where infection was highly suspected, strategies to reduce theduration of antibiotics based on the biomarker procalcitonin shortened antibiotic therapy in the intensive care unit (ICU) and did not appear to worsen mortality (though these studies were small) [10-12].

In our study, prompting was also associated with shorter central venous catheter duration and increased ventilator-free days [6]. Previous studies have reported that interventions to reduce the duration of mechanical ventilation or central venous catheters are associated with improved outcomes [13-17].

It is important to understand which of these processes of care improvements contributed to lower mortality in the group that received prompting. The objective of the present study was to perform post hoc exploratory analyses to examine if differences in care components addressed by the checklist—specifically empiric antibiotic duration, mechanical ventilation, and central venous catheter duration—were mediators of the observed difference in hospital mortality between the intervention group that received prompting and the unprompted control group. In addition, we sought to investigate the relationship between empiric antibiotic duration and mortality independent of the effect of group assignment.

## Methods

### Study design

Some of the details of the methods used in this study have been previously reported [6]. In brief, we conducted an investigation among two medical ICU (MICU) teams at Northwestern Memorial Hospital, a tertiary care urban hospital affiliated with an academic medical center. This study was approved by the Northwestern University Institutional Review Board with a waiver of consent (study number: STU00013313). The MICU is a closed unit staffed by two teams, each with an independent patient census. The two teams admit patients on alternating days and are comprised of one pulmonary/critical care attending physician, one fellow, one pharmacist, and several residents and interns. Attendings and fellows have weekday rotations of 1–4 weeks, frequently with different weekend coverage. Physicians were arbitrarily assigned to the two MICU teams prior to and without knowledge of the study. At the onset of the study, which team was assigned to prompting was randomly chosen—team membersdid not choose to be part of the prompted or control teams.

A MICU daily rounding checklist was instituted in March 2009 for both teams as a quality improvement tool. It was designed to address process of care issues not directly related to the primary illness and therefore potentially overlooked.

### Intervention

From June 25 to September 15, 2009, one MICU team (prompted team) was assigned to receive an intervention that consisted of prompting during daily rounds for all patients under their care. A non-care providing resident physician (the prompter) initiated discussion with the attending physician on the prompted team during daily bedside rounds if any of the following six parameters under investigation were overlooked: empiric antibiotic utilization, mechanical ventilation weaning, central venous catheters (CVCs), Foley urinary catheters, and deep vein thrombosis (DVT) and stress ulcer prophylaxis. For example, if the rounding team failed to discuss the utilization or discontinuation of empiric antibiotics, the prompter would ask, “The patient has been on [empiric antibiotic] for [X]days. Do you want to continue it?” The prompters had no patient care responsibilities; there was no contact between the prompters and any patient. The second MICU team was unprompted, but continued to have the identical checklist available, and served as a concurrent control.

The following patients were excluded from analysis: 1) patients physically located in adifferent ICU for more than the first 72 hours of their ICU stay, 2) patients transferred from a different ICU service (e.g. surgical ICU), and 3) patients transferred to a different ICU service within 12 hours of MICU admission. Also, only the first MICU admission was included for patients admitted more than once without intervening hospital discharge [18,19]. Any patient admitted to the prompted team was included regardless of whether the prompter was present during their ICU stay. Prompting began during the first rounds after a patient’s MICU admission and continued daily (whenever the prompter was present) until MICU discharge.

### Measurement of care processes and outcomes

Empiric antibiotics were defined as antibiotics administered without culture-documented infection. Central venous catheters excluded hemodialysis catheters and peripherally inserted central catheters (PICCs). Hospital mortality was collected from the hospital’s electronic health record. In order to perform risk adjustment, data necessary to calculate Acute Physiology and Chronic Health Evaluation (APACHE) IV predicted hospital mortality and ICU LOS within the first 24 hours of MICU admission were collected and scored retrospectively [18,19]. These data were collected by personnel blinded to group assignment.

Care process duration for empiric antibiotics and central venous catheters was categorized into 1–3days (Category 1), 4–6days (Category 2), and ≥7days (Category 3). A 72 hour cutoff for Category 1 was chosen because this interval is shorter than the threshold of increased risk of ventilator-associated pneumonia, central line-associated bloodstream infection, and urinary catheter-related infection [20-22]; approximately the time point at which de-escalation of empiric antibiotics is considered [23]; and approximately when final bacterial culture results become available in our laboratory. To test whether our findings were sensitive to the empiric antibiotic categories we chose, we performed an additional analysis with empiric antibiotic duration categories of 1–3days, 4–5days, and ≥6days.

### Analytic plan

Three care processes—empiric antibiotic duration, days alive and ventilator-free within the first 28 days after initiation of mechanical ventilation, and central venous catheter duration—differed significantly between the prompted and control groups [6]. Each process of care was analyzed separately. For the analysis of each care process, patients were excluded who were never eligible to be prompted for that process (e.g. patients who never received antibiotics never triggered prompting to consider stopping antibiotics).

To perform a mediating effects analysis, we constructed a logistic regression base model containing group assignment and APACHE IV predicted hospital mortality. We then added categorical variables for empiric antibiotic duration to the model to determine their mediating effect on the adjusted odds ratio of death for the prompted group variable (i.e. the degree to which the adjusted odds ratio for death for the prompted group changed with the addition of empiric antibiotic categories). In addition, we constructed a separate logistic regression model that did not include the group variable to examine whether empiric antibiotic duration categories were independent predictors of risk-adjusted mortality. The category representing the shortest duration of antibiotics was used as a reference for each mediating effects regression model described above.

Similar models were created to examine the separate mediating effect of 1) mechanical ventilation, and 2) central venous catheters on the group odds ratio of death. For patients who received mechanical ventilation at any time during their ICU admission, we determined whether prompting was associated with an increased proportion of patients liberated from mechanical ventilation.

Previously, we demonstrated a significant increase in the proportion of eligible prompted group patients who received DVT and stress ulcer prophylaxis [6]. However, the high proportion of patients in both groups who received these therapies made it difficult to include them in a mediating effects model. Prompting did not significantly reduce Foley urinary catheter duration, so this too was not explored as a potential mediator.

We determined three characteristics of experience for each attending physician: number of “on-service” MICU days from 6/25/08-6/24/09 (one full year prior to the prompting intervention), number of years since pulmonary/critical care fellowship completion, and a model that included both of these variables. We constructed logistic regression models by weighting the above variables based on the percentage each patient was exposed to each attending during their MICU stay.

We used a χ^2^ test to compare categorical variables. For logistic regression models, odds ratios (OR) are reported with 95% confidence intervals (CIs). All tests were two-tailed, and a *P* value of <0.05 was considered significant. Analyses were performed using SAS (version 9.2, Cary, NC).

## Results

Two hundred sixty-five patients were included in the study, 140 in the prompted group and 125 in the control group. Baseline characteristics were not different between the prompted and control groups (Table 1). As previously reported, a prompter was present on 67.9% of prompted group daily rounds. Overall, prompting was required on 64.7% of patient-days. Empiric antibiotic prompting was required on 36.3% patient-days, mechanical ventilation prompting was required on 14.1% patient-days, and central venous catheter prompting was required on 25.7% patient-days [6]. Process of care and clinical outcomes also have been previously reported [6]. Attending experience did not differ between the prompted and control teams, and none of the attending variables were predictors of hospital mortality.

**Table 1:** Baseline characteristics of study patients

**Characteristic**	**Prompted (N=140)**	**Control (N=125)**	***P*value**
Age (years), mean (SD)	58.5 (17.8)	57.3 (17.8)	0.60
Gender (male), no. (%)	69 (49.3)	51 (40.8)	0.17
Race, no. (%)			
White	71 (50.7)	69 (55.2)	
African American	47 (33.6)	42 (33.6)	0.54
Hispanic/Other	22 (15.7)	14 (11.2)	
Mechanical Ventilation, no. (%)	36 (28.8)	41 (29.3)	0.93
APACHE IV predicted hospital mortality, no. (%)	31.1 (22.2)	27.2 (21.7)	0.86

### Empiric antibiotics

One hundred eighty-three patients in this study received empiric antibiotics on at least one day during their MICU admission. In this sub-group of patients who received empiric antibiotics, there were also no differences in baseline characteristics. The percentage of patients who received empiric antibiotics was not statistically different in the prompted and control groups (64.3% vs. 74.4%, *P=*0.075). As previously reported, prompting to consider empiric antibiotic discontinuation was required on 36.3% of patient-days, while median empiric antibiotic duration was reduced to two days in the prompted group compared to three days in the control group [6].

As empiric antibiotic duration increased, unadjusted hospital mortality concomitantly increased (*P* for trend<0.001) (Table 2). Prompting was associated with reduced risk-adjusted odds of death in all patients who received empiric antibiotics (OR 0.41, 95% CI 0.18-0.92, *P=*0.032), and in patients who received empiric antibiotics for 1–3 days (OR 0.17, 95% CI 0.04-0.75, *P=*0.019). There were no statistically significant differences in mortality between the prompted and control groups in patients who received empiric antibiotics for 4–6 or ≥7days, respectively.

**Table 2:** Hospital mortality according to duration of empiric antibiotics (N=183)

**Duration of empiric antibiotics (days)**	**Hospital mortality, deaths/N (%)**^**a**^
1-3	17/121 (14.0)
4-6	8/29 (27.6)
≥7	14/33 (42.4)

^a^*P* for trend<0.001.

In the baseline mortality model (adjusted only for APACHE IV score), the adjusted odds ratio of death was 0.41 (95% CI 0.18-0.93, *P=*0.032) for patients in the prompted group (Table 3). When empiric antibiotic duration categories were added to this model, the mortality difference was attenuated (OR 0.50, 95% CI 0.21-1.2, *P=*0.11). The percent difference in the adjusted OR of death between the baseline model and full model explained by the addition of empiric antibiotic categories was 15.2%. Thus, 15.2% of the risk-adjusted mortality reduction associated with prompting can be accounted for by the reduction of empiric antibiotic duration with prompting.

**Table 3:** Multivariable analysis of the effect of intervention group and empiric antibiotics on hospital mortality

	**OR (95% CI)**	***P *value**
***Intervention effect base model:***		
Prompted group	0.41 (0.18-0.92)	0.032
APACHE IV predicted mortality	47.8 (10.3-222)	<0.001
***Empiric antibiotics mediating effect model:***^***a***^		
Prompted group	0.50 (0.21-1.2)	0.11
APACHE IV predicted mortality	37.0 (7.6-180)	<0.001
Empiric antibiotics 4–6days	2.1 (0.76-6.0)	0.15
Empiric antibiotics ≥7days	2.5 (0.92-6.6)	0.074
***Independent empiric antibiotics model:***^***a***^		
APACHE IV predicted mortality	30.5 (6.6-140)	<0.001
Empiric antibiotics 4–6days	2.3 (0.81-6.3)	0.12
Empiric antibiotics ≥7days	3.1 (1.2-8.0)	0.019

^a^ Category 1 (empiric antibioticduration 1–3days) used as reference

OR: Odds ratio ofdeath. CI: confidence interval.

In the empiric antibiotics-only model, we found that empiric antibiotic duration of ≥7days was an independent predictor of risk-adjusted mortality (OR 3.1, 95% CI 1.2-8.0, *P=*0.019), while empiric antibiotic duration of 4–6days increased the odds of death but was not statistically significant (Table 3). The results were similar in sensitivity analyses using alternative categorization schemes for empiric antibiotic duration.

### Mechanical ventilation

Seventy-seven patients (29% of the total study population) received mechanical ventilation through an endotracheal or tracheotomy tube during their MICU admission. As previously reported, prompting to consider performing a spontaneous breathing trial was required on 14.1% of patient-days, while median ventilator-free days was higher in the prompted group compared to control (22 [14–26]days vs. 16 [0–21.5]days, P=0.028) [6].

There was no statistically significant difference in the percentage of patients in the prompted group who were liberated from mechanical ventilation compared to control (78.0% vs. 66.7%, P=0.31). After adjustment for APACHE IV severity, the odds ratio (95% CI) of being liberated from mechanical ventilation in the prompted group was 1.7 (0.5-5.7, *P=*0.38). The number of mechanically ventilated patients was too small to conduct formal mediation analyses to determine to what degree the higher rate of liberation from mechanical ventilation explained the overall benefit of prompting.

### Central venous catheters

One hundred three patients had a central venous catheter in place during their MICU admission, including CVCs inserted prior to MICU admission. The percentage of patients who received central venous catheters was not statistically different in the prompted and control groups (39.3 vs. 38.4%, *P=*0.88). As previously reported, prompting to consider removing a CVC was required on 25.7% of patient-days, while median CVC duration was shortened to three days in the prompted group compared to five days in the control group [6].

As central venous catheter duration increased, a corresponding increase in the hospital mortality rate was seen (CVC duration 1–3days, 15.8%; CVC duration 4–6days, 23.8%; and CVC duration ≥7days, 36.0%, respectively; *P* for trend=0.043). In the base model, among all patients with a CVC, the prompted group had a risk-adjusted mortality odds ratio (95% CI) of 0.48 (0.17-1.4, *P=*0.17). In the model that included central venous catheter duration categories, the mortality reduction in the intervention group was slightly attenuated, OR to 0.54 (0.18-1.6, *P=*0.27). Neither model was statistically significant.

## Discussion

Our exploratory analyses suggest that the lower risk-adjusted mortality seen in the intervention group that received prompting was due to multiple improvements in processes of care, each of which may have had small, incremental contributions to lowering mortality. Reducing empiric antibiotic duration appears to have had the largest individual effect, but reducing the duration of central venous catheters also may have contributed. While the higher rate of liberating patients from mechanical ventilation in the intervention group likely contributed to lower mortality, we were not able to assess the magnitude of this in formal mediation analyses because of the small number of mechanically ventilated patients.

There are two central findings related to empiric antibiotic utilization. First, a strategy of prompting that shortened the duration of empiric antibiotics may account for some of the mortality benefits seen with the prompting intervention. To our knowledge, this is the first report that an intervention strategy that reduces empiric antibiotic duration may mediate a reduction in risk-adjusted mortality. Concern for clinical deterioration after discontinuing empiric antibiotics leads many providers to continue them even in the face of documented negative microbial cultures [9,24-26]. Our study contradicts this concern and suggests that strategies that reduce empiric antibiotic overuse may prevent potentially harmful consequences.

This conclusion must be interpreted cautiously, becauseduration of empiric antibiotics may be a marker of ongoing or worsening severity of illness. At a minimum, however, shortening empiric antibiotic duration did not worsen mortality. This result is consistent with prior research that has shown that when infection was highly suspected, shortening antibiotic therapy based on procalcitonin measurement did not increase mortality [10,11].

In addition, we demonstrated that prolonged empiric antibiotic duration is an independent predictor of risk-adjusted mortality. This finding supports prior research that has shown an association between prolonged use of empiric antibiotics and mortality [8,9]. In particular, Aarts and coworkers reported that prolonged empiric antibiotic therapy (>4days) in patients without nosocomial infection was associated with an almost 4-fold increase in the age- and risk-adjusted odds of death that almost reached statistical significance [9].

The second main finding is that a large, unexplained association between prompting and mortality remained after adjusting for empiric antibiotic utilization. There may be several reasons for this. Significant synergistic effects among empiric antibiotics, mechanical ventilation, and central venous catheters may exist, given these processes’ closely related clinical utilization. Also, we had imperfect measures of potential mediating care processes. For example, the duration of empiric antibiotics may not capture all possible information about the effect of empiric antibiotics on the prevention of antibiotic-related side effects, such as antibiotic resistance, that may then contribute to mortality. In addition, the study definition of what constituted an empiric antibiotic was intentionally broad (antibiotics administered without culture-documented infection). Had a more narrow definition been employed, empiric antibiotic duration may possibly have had a greater mediating effect.

Moreover, we do not know which specific reductions in empiric antibiotics were attributable to prompting, since an individual prompt may have impacted other processes of care or the empiric antibiotic management for other patients [6]. As a result, prompting may lead to other unmeasured changes in physician behavior. For example, prompting to discontinue empiric antibiotics for an individual patient may have had a learned behavioral effect on the caregiver to consider antibiotic changes in other patients prior to further prompting. Finally, the full effect of prompting on mortality may be the result of numerous small, incremental improvements that escaped measurement. These issues deserve further study in large, multi-center trials that are powered not only for mortality or other clinical outcomes, but also adequate to analyze causal mechanisms more completely.

Our study had several potential limitations, some described previously [6]. It was based on a small cohort conducted at a single institution, limiting statistical power in these exploratory analyses. In particular, analysis of mechanical ventilation and central venous catheter analyses was difficult due to low statistical power. In addition, the APACHE IV prediction model employed for risk adjustment is based on the first 24 hours of ICU admission [18,19]. Ongoing severity-of-illness, which wedid not measure, could have affected the delivery of the processes of care that we investigated.

We did not adjust variances for clustering of patients within clinicians. Since the treatments and tests received by patients cared for by the same physician can be correlated, lack of adjustment for clustering can overestimate tests of statistical significance. However, as is typical of critical care practice, patients in our study were frequently cared for by multiple physicians (and other care providers)during their ICU stay. There is no established method to account for clustering when there are multiple providers per patient and the configuration of providers changes across patients (i.e. there are not fixed teams). The fact that multiple physicians cared for patients during their stay decreases the influence of any individual physician, so it is unlikely that adjustment for clustering would significantly alter our results.

It is possible that if practice patterns differed by team, this could have confounded the results of the study. This issue was addressed in four ways. First, we randomly assigned the prompted and control teams after physician clinical schedules were published. Second, there were no differences between the two teams in attending physician experience or the effect of attending experience on mortality. Third, no other structure or process improvements affecting the MICU teams were made during the study. Finally, attending physicians were only “on-service” for one to two weeks, with different attending physicians covering the weekends. Due to this frequent rotation, many patients were likely treated by more than one attending, further diluting any practice pattern confounding. The possibility that some unmeasured confounding exists remains as a limitation to this study.

## Conclusions

In summary, our findings support the hypothesis that some of the favorable mortality effects of a prompting intervention were due to reduced empiric antibiotic duration. However, much of the effect of prompting on mortality remained unexplained. This could suggest that multiple improvements in, and synergy between, processes of care may have had small, incremental contributions to lowering mortality. We believe the data warrant future investigations of whether strategies that specifically reduce empiric antibiotic duration and improve other processes of care lead to improved ICU outcomes.

## Abbreviations

APACHE, Acute Physiology And Chronic Health Evaluation; CVC, Central Venous Catheter; DVT, Deep Vein Thrombosis; ICU, Intensive Care Unit; LOS, Length Of Stay; MICU, Medical Intensive Care Unit; OR, Odds Ratio.

## Competing interests

The authors declare that they have no competing interests.

## Authors’ contributions

CW conceived the study, participated in the design of the study, participated in performing the statistical analysis, and drafted the manuscript. SP participated in the design of the study, participated in performing the statistical analysis, and helped draft the manuscript. RW helped conceive the study, participated in the design of the study, and helped draft the manuscript. DB participated in the design of the study, participated in performing the statistical analysis, and helped draft the manuscript. All authors read and approved the final manuscript.

## Pre-publication history

The pre-publication history for this paper can be accessed here:

http://www.biomedcentral.com/1472-6963/12/198/prepub

## References

[B1] DuBose JJ Inaba K Shiflett A Trankiem C Teixeira PG Salim A Rhee P Demetriades D Belzberg H Measurable outcomes of quality improvement in the trauma intensive care unit: the impact of a daily quality rounding checklist J Trauma 2008 64 22 27 10.1097/TA.0b013e318161b0c8 18188094

[B2] Dubose J Teixeira PG Inaba K Lam L Talving P Putty B Plurad D Green DJ Demetriades D Belzberg H Measurable outcomes of quality improvement using adaily quality rounds checklist: one-year analysis in a trauma intensive care unit with sustained ventilator-associated pneumonia reduction J Trauma 2010 69 855 860 10.1097/TA.0b013e3181c4526f 20032792

[B3] Walsh TS Dodds S McArdle F Evaluation of simple criteria to predict successful weaning from mechanical ventilation in intensive care patients Br J Anaesth 2004 92 793 799 10.1093/bja/aeh139 15121724

[B4] Haynes AB Weiser TG Berry WR Lipsitz SR Breizat AH Dellinger EP Herbosa T Joseph S Kibatala PL Lapitan MC Merry AF Moorthy K Reznick RK Taylor B Gawande AA A surgical safety checklist to reduce morbidity and mortality in a global population N Engl J Med 2009 360 491 499 10.1056/NEJMsa0810119 19144931

[B5] Byrnes MC Schuerer DJ Schallom ME Sona CS Mazuski JE Taylor BE McKenzie W Thomas JM Emerson JS Nemeth JL Bailey RA Boyle WA Buchman TG Coopersmith CM Implementation of a mandatory checklist of protocols and objectives improves compliance with a wide range of evidence-based intensive care unit practices Crit Care Med 2009 37 2775 2781 10.1097/CCM.0b013e3181a96379 19581803

[B6] Weiss CH Moazed F McEvoy CA Singer BD Szleifer I Amaral LA Kwasny M Watts CM Persell SD Baker DW Sznajder JI Wunderink RG Prompting physicians to address adaily checklist and process of care and clinical outcomes: a single-site study Am J Respir Crit Care Med 2011 184 680 686 10.1164/rccm.201101-0037OC 21616996 PMC3208596

[B7] Niederman MS Soulountsi V De-escalation therapy: is it valuable for the management of ventilator-associated pneumonia? Clin Chest Med 2011 32 517 534 10.1016/j.ccm.2011.05.009 21867820

[B8] Singh N Rogers P Atwood CW Wagener MM Yu VL Short-course empiric antibiotic therapy for patients with pulmonary infiltrates in the intensive care unit. A proposed solution for indiscriminate antibiotic prescription Am J Respir Crit Care Med 2000 162 505 511 10934078 10.1164/ajrccm.162.2.9909095

[B9] Aarts MA Brun-Buisson C Cook DJ Kumar A Opal S Rocker G Smith T Vincent JL Marshall JC Antibiotic management of suspected nosocomial ICU-acquired infection:does prolonged empiric therapy improve outcome? Intensive Care Med 2007 33 1369 1378 10.1007/s00134-007-0723-y 17558493

[B10] Hochreiter M Kohler T Schweiger AM Keck FS Bein B von Spiegel T Schroeder S Procalcitonin to guideduration of antibiotic therapy in intensive care patients: a randomized prospective controlled trial Crit Care 2009 13 R83 10.1186/cc7903 19493352 PMC2717450

[B11] Stolz D Smyrnios N Eggimann P Pargger H Thakkar N Siegemund M Marsch S Azzola A Rakic J Mueller B Tamm M Procalcitonin for reduced antibiotic exposure in ventilator-associated pneumonia: a randomised study Eur Respir J 2009 34 1364 1375 10.1183/09031936.00053209 19797133

[B12] Kopterides P Siempos II Tsangaris I Tsantes A Armaganidis A Procalcitonin-guided algorithms of antibiotic therapy in the intensive care unit: a systematic review and meta-analysis of randomized controlled trials Crit Care Med 2010 38 2229 2241 10.1097/CCM.0b013e3181f17bf9 20729729

[B13] Feng Y Amoateng-Adjepong Y Kaufman D Gheorghe C Manthous CA Age,duration of mechanical ventilation, and outcomes of patients who are critically ill Chest 2009 136 759 764 10.1378/chest.09-0515 19736189

[B14] Kress JP Pohlman AS O'Connor MF Hall JB Daily interruption of sedative infusions in critically ill patients undergoing mechanical ventilation N Engl J Med 2000 342 1471 1477 10.1056/NEJM200005183422002 10816184

[B15] Rumbak MJ Newton M Truncale T Schwartz SW Adams JW Hazard PB A prospective, randomized, study comparing early percutaneousdilational tracheotomy to prolonged translaryngeal intubation (delayed tracheotomy) in critically ill medical patients Crit Care Med 2004 32 1689 1694 10.1097/01.CCM.0000134835.05161.B6 15286545

[B16] Berenholtz SM Pronovost PJ Lipsett PA Hobson D Earsing K Farley JE Milanovich S Garrett-Mayer E Winters BD Rubin HR Dorman T Perl TM Eliminating catheter-related bloodstream infections in the intensive care unit Crit Care Med 2004 32 2014 2020 10.1097/01.CCM.0000142399.70913.2F 15483409

[B17] Pronovost P Needham D Berenholtz S Sinopoli D Chu H Cosgrove S Sexton B Hyzy R Welsh R Roth G Bander J Kepros J Goeschel C An intervention todecrease catheter-related bloodstream infections in the ICU N Engl J Med 2006 355 2725 2732 10.1056/NEJMoa061115 17192537

[B18] Zimmerman JE Kramer AA McNair DS Malila FM Acute Physiology and Chronic Health Evaluation (APACHE) IV: hospital mortality assessment for today's critically ill patients Crit Care Med 2006 34 1297 1310 10.1097/01.CCM.0000215112.84523.F0 16540951

[B19] Zimmerman JE Kramer AA McNair DS Malila FM Shaffer VL Intensive care unit length of stay: Benchmarking based on Acute Physiology and Chronic Health Evaluation (APACHE) IV Crit Care Med 2006 34 2517 2529 10.1097/01.CCM.0000240233.01711.D9 16932234

[B20] Garnacho-Montero J Aldabo-Pallas T Palomar-Martinez M Valles J Almirante B Garces R Grill F Pujol M Arenas-Gimenez C Mesalles E Escoresca-Ortega A de Cueto M Ortiz-Leyba C Risk factors and prognosis of catheter-related bloodstream infection in critically ill patients: a multicenter study Intensive Care Med 2008 34 2185 2193 10.1007/s00134-008-1204-7 18622596

[B21] Hammarskjold F Wallen G Malmvall BE Central venous catheter infections at a county hospital in Sweden: a prospective analysis of colonization, incidence of infection and risk factors Acta Anaesthesiol Scand 2006 50 451 460 10.1111/j.1399-6576.2006.00974.x 16548857

[B22] vander Kooi TI de Boer AS Mannien J Wille JC Beaumont MT Mooi BW vanden Hof S Incidence and risk factors ofdevice-associated infections and associated mortality at the intensive care in the Dutch surveillance system Intensive Care Med 2007 33 271 278 10.1007/s00134-006-0464-3 17146632

[B23] Niederman MS The importance ofde-escalating antimicrobial therapy in patients with ventilator-associated pneumonia Semin Respir Crit Care Med 2006 27 45 50 10.1055/s-2006-933673 16508881

[B24] Namias N Harvill S Ball S McKenney MG Salomone JP Sleeman D Civetta JM Empiric therapy of sepsis in the surgical intensive care unit with broad-spectrum antibiotics for 72 hoursdoes not lead to the emergence of resistant bacteria J Trauma 1998 45 887 891 10.1097/00005373-199811000-00008 9820698

[B25] Heyland DK Cook DJ Marshall J Heule M Guslits B Lang J Jaeschke R The clinical utility of invasivediagnostic techniques in the setting of ventilator-associated pneumonia Canadian Critical Care Trials Group Chest 1999 115 1076 1084 10.1378/chest.115.4.1076 10208211

[B26] Sole Violan J Fernandez JA Benitez AB Cardenosa Cendrero JA Rodriguezde Castro F Impact of quantitative invasive diagnostic techniques in the management and outcome of mechanically ventilated patients with suspected pneumonia Crit Care Med 2000 28 2737 2741 10.1097/00003246-200008000-00009 10966244

